# Errata

**DOI:** 10.36660/abc.20240279

**Published:** 2024-05-07

**Arq Bras Cardiol. 2018; 110(1):44-51**

No Artigo Original "Efeitos Protetores Induzidos pela Melatonina nos Cardiomiócitos Contra Lesões de Reperfusão Parcialmente Através da Modulação Dde IP3R e SERCA por Meio da Ativação de ERK1", com número de DOI: 10.5935/abc.20180008, publicado no periódico Arquivos Brasileiros de Cardiologia, Arq Bras Cardiol. 2018; 110(1):44-51, na página 49, corrigir a Figura 5A pela Figura 5A apresentada abaixo.

**Figure:**
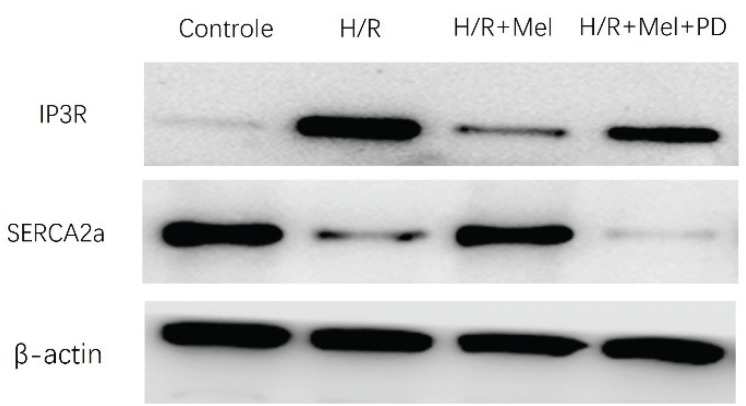


**Arq Bras Cardiol. 2022; 118(6):1150-1152**

No Artigo Imagem "Valor do ^18^F-FDG PET/CT no Diagnóstico e Avaliação de Resposta ao Tratamento da Miocardite Lúpica", com número de DOI: https://doi.org/10.36660/abc.20210523, publicado no periódico Arquivos Brasileiros de Cardiologia, Arq Bras Cardiol. 2022; 118(6):1150-1152, na página 1150, corrigir o nome da autora Mariana Feitosa Ramalho Galvão para Marina Feitosa Ramalho Galvão.

**Arq Bras Cardiol. 2024; 121(4):e20230386**

No Artigo Original "Fatores Associados aos Custos do Tratamento no Primeiro Ano após Implantes Iniciais ou Trocas de Geradores de Pulsos de Marcapassos Cardíacos", com número de DOI: https://doi.org/10.36660/abc.20230386, publicado no periódico Arquivos Brasileiros de Cardiologia, Arq Bras Cardiol. 2024; 121(4):e20230386, na página 1, corrigir o nome do autor Lucas Bassoli de Oliveira Alves para autor Lucas Bassolli de Oliveira Alves.

