# Supplementary material for: Errata
Source: Arq Bras Cardiol. 2024 May 7;121(5):e20240279. [Article in Portuguese] doi: 10.36660/abc.20240279 (PMC11081409; doi:10.36660/abc.20240279)
Supplement: Translation

# Translation: Erratum

**Arq Bras Cardiol. 2018; 110(1):44-51**

In the Original Article "Melatonin-Induced Protective Effects on Cardiomyocytes Against Reperfusion Injury Partly Through Modulation of IP3R and SERCA2a Via Activation of ERK1", with DOI: 10.5935/abc.20180008, published in the journal Arquivos Brasileiros de Cardiologia, Arq Bras Cardiol. 2018; 110(1):44-51, on page 49, correct Figure 5A to Figure 5A shown below.

**Arq Bras Cardiol. 2022; 118(6):1150-1152**

In the Image Article "Value of^18^F-FDG PET/CT in the Diagnosis and Assessment of Response to Treatment of Lupus Myocarditis", with DOI: https://doi.org/10.36660/abc.20210523, published in the journal Arquivos Brasileiros de Cardiologia, Arq Bras Cardiol. 2022; 118(6):1150-1152, on page 1150, correct the name of the author Mariana Feitosa Ramalho Galvão to Marina Feitosa Ramalho Galvão.

**Arq Bras Cardiol. 2024; 121(4):e20230386**

In the Original Article "Factors Associated with the Treatment Costs within the First Year after Pacemaker Implantation or Pulse Generator Replacement", with DOI: https://doi.org/10.36660/abc.20230386, published in the journal Arquivos Brasileiros de Cardiologia, Arq Bras Cardiol. 2024; 121(4):e20230386i, on page 1, correct the name of the author Lucas Bassoli de Oliveira Alves to Lucas Bassolli de Oliveira Alves.
